# Nutrient Intakes among Brazilian Children Need Improvement and Show Differences by Region and Socioeconomic Level

**DOI:** 10.3390/nu14030485

**Published:** 2022-01-22

**Authors:** Andrea S. Anater, Joel C. Hampton, Tássia do Vale Cardoso Lopes, Eliana B. Giuntini, Vanessa C. Campos, Lisa J. Harnack, Julia M. Lorenzana Peasley, Alison L. Eldridge

**Affiliations:** 1 RTI International, Research Triangle Park, NC 27709, USA; jhampton@rti.org; 2 Food Research Center—FoRC, Centro de Pesquisa em Alimentos, Cidade Universitária, University of São Paulo, São Paulo 05508-080, Brazil; tassia.dovale@alumni.usp.br (T.d.V.C.L.); elibi@alumni.usp.br (E.B.G.); 3 Nestlé Institute of Health Sciences, Nestlé Research, 1000 Lausanne, Switzerland; VanessaCaroline.Campos@rdls.nestle.com (V.C.C.); alison.eldridge@rdls.nestle.com (A.L.E.); 4 Nutrition Coordinating Center, School of Public Health, University of Minnesota, Minneapolis, MN 55454, USA; harna001@umn.edu (L.J.H.); peas0027@umn.edu (J.M.L.P.)

**Keywords:** Brazil, nutrient intake, dietary intake, nutritional epidemiology, children

## Abstract

Brazil is the most populous country in South America. Using 24 h dietary data, we compared the nutrient intakes of 4–13-year-olds to reference values and tested for regional and socioeconomic (SES) differences. A considerable proportion reported intakes below the Estimated Average Requirements (EAR) for vitamins E (78.1%, 96.5%), D (100% for both), and calcium (80.5%, 97.7%) for 4–8 and 9–13-year-olds, respectively. Few exceeded Adequate Intakes (AI) for potassium or fiber. Older children reported greater inadequacies and, while there was regional variability, patterns of inadequacy and excess tended to be similar. For vitamin C, the percent of children below EAR in the Northeast and Southeast was lower than in the South. Most children, regardless of SES, had energy intakes within the Acceptable Macronutrient Distribution Ranges (AMDRs) for carbohydrates and protein. Over a quarter reported total energy from fat less than the AMDR, and inversely associated with SES (low 50.9%, moderate 26.0%, and high 15.0%), but also exceeding the percentage of energy recommendation for saturated fat, increasing with SES (low 18.1%, moderate 38.9%, and high 48.8%). The contrast observed between the diets of young Brazilians and recommendations underscores the need for individual and regional environmental interventions to promote healthier dietary patterns.

## 1. Introduction

Brazil is by far the largest and most populous country in South America and the fifth largest in the world, with diverse diets that vary by region, reflecting the country’s mix of native and immigrant populations. According to the United Nations, in 2020 Brazil’s population was around 213 million people [1]. While beans and rice are dietary staples across the country, more manioc flour is consumed in the North and Northeast, whereas more rice and potatoes are consumed in the South and Southeast. Milk consumption is reportedly low in the North, whereas soft drink consumption is highest in the Southeast [2]. Other studies have reported lower intakes of fruits and vegetables in the Northeast and higher intakes of sweets in the South [3]. Specifically, among adolescents, beans were the second most consumed food in the Southeast and Northeast regions, whereas in the South, bread and beef preceded beans in consumption; rice was the most consumed food in all three regions. The South region showed the highest prevalence of carbonated soft drinks and milk consumption. While vegetables were reportedly consumed most often in the Southeast, they were not among the top five most consumed foods in any of the three regions [4]. Differences in food availability, habits, and preferences may influence the nutrient intakes by region.

Studies on nutrient adequacy in the diets of Brazilian children have focused more on older children and adolescents, and most do not consider differences by region. Some publications address the adequacy of nutrients in the diets of Brazilian adolescents and children, but most do not consider differences in younger children by region and socioeconomic level. The last Brazilian national nutrition survey was conducted in 2008–2009 and included a national sample of individuals ≥10 years of age [5]. In 2007, a multicenter cross-sectional survey was conducted with 2–6-year-olds from nine cities across the country [6]. The Brazilian Study of Cardiovascular Risks in Adolescents, conducted in 2013–2104, reported on both nutrient intakes and food groups. Although the findings from these studies point to some nutritional inadequacies [4], they do not all report on macro- and micronutrients, nor do they cover the full age range of young, school-aged children.

In the absence of data on nutrient intakes, several studies have associated overall diet quality scores with socio-economic status or age. Higher dietary diversity and higher nutrient adequacy scores were reported in older adolescents and adults from higher vs. lower socio-economic groups [7]. Similarly, the Healthy Eating Index (HEI) scoring metric has been used in Brazil to evaluate adult diet quality; higher HEI scores were reported by those with higher income levels [8]. Similarly, dietary pattern analysis in adolescents showed differences by socio-economic level, with higher-income adolescents eating more of a Western-type diet and those in the lower SES groups eating a more traditional Brazilian diet [9]. Another study among schoolchildren showed that children <10 years of age had higher intakes of a “healthy” dietary pattern than adolescents ≥10 years of age [10].

The changes in the dietary guidelines in Brazil have shifted the focus from food-based guidelines to meet nutrient adequacy [11] to guidelines based on reducing the consumption of processed foods [12]. Difficulties have been identified in interpreting studies that rely on degree of processing to assess dietary adequacy in children [13], and this classification system leaves unanswered questions about the nutrient adequacy of child diets for optimal development. The purpose of this study was to estimate the total usual dietary intakes for energy and most nutrients among Brazilian children aged 4–13 years of age and to assess regional and sociodemographic differences.

## 2. Materials and Methods

The Brazil Kids Nutrition and Health Study is a cross-sectional survey of 4–13-year-old children conducted in the following three most populated regions of Brazil: Northeast, Southeast, and South to:

1. Provide robust public health data about the dietary intakes of children aged 4–13 years;
2. Develop data to enable further research about the potential associations between sociodemographic and lifestyle variables and the dietary patterns and nutrient intakes of children; and
3. Identify areas in the diet where children and potential subpopulations may benefit from targeted interventions.

Data were collected by trained field workers in homes from October 2019 to February 2020. Parents or caregivers provided informed consent. The study was conducted in accordance with the Declaration of Helsinki, and the protocol, procedures, and all instruments were reviewed and approved by Institutional Review Boards at RTI International (Research Triangle Park, NC, USA) and University of São Paulo (São Paulo, Brazil). In addition, we obtained study approval from CONEP (*Comissão Nacional de Ética em Pesquisa*). Brazil’s National Council for Ethics in Research (CONEP) is the central statutory body responsible for registering, auditing and accrediting institutional ethics committees and is the advisory body for the Brazilian Ministry of Health.

### 2.1. Sample

Brazil is officially divided into five large geographic regions. For practical and logistical reasons (e.g., boat travel required to reach villages in Amazonian rainforest) children 4–13 years of age residing in the three most populated regions—Northeast, Southeast, and South—were randomly selected for inclusion. Combined, these three regions account for 83.3% of the Brazilian population [14]. We used a multistage, probability design to select a representative sample from the three regions. To ensure maximum randomization, the sample was stratified and included four stages of selection. First, the three regions’ populations were stratified into nine geographical areas composed of groupings of states (three per region). In the Northeast, we grouped contiguous states into three strata with approximately equal numbers of children. In the Southeast region, we assigned the cities of Rio de Janeiro and São Paulo to their own stratum with the remaining two states placed in a third stratum. Each of the three states in the South composed their own stratum. Within each of these strata, we randomly selected one state for inclusion, with probability proportional to the number of 4–13-year-olds. Next, Brazilian sectors, similar to U.S. Census block groups, were selected with probability proportional to the estimated number of 4–13-year-olds. Supplemental sectors were chosen to be used as needed to complement data collection in the original sectors or to serve as replacement locations following the same rules for selection. Population estimates were based on the 2008 National Household Sample Survey (PNAD), conducted by the Brazilian Institute of Geography and Statistics (IBGE), Brazilian Statistics Bureau. An adjustment to the population totals was applied to account for the age of the data. Within each selected sector, we ordered households to be recruited by randomizing a starting point and then field interviewers conducted a random walk through the sector, adhering to a strict set of rules that specified household selection according to estimated number of children within the sector. Finally, within eligible households, one child aged 4–13 years was randomly selected for study participation. Data were collected for 983 children from the following three regions: Northeast (*n* = 334), South (*n* = 311) and Southeast (*n* = 318). The overall study response rate was calculated as the product of the household response rate to the screener (70.4%) and individual response rate (98.4%). The overall study response rate was 69.3%.

### 2.2. Instrument Design

The instrument consisted of the following five modules: (1) a household screener; (2) age-specific dietary questionnaires and 24 h dietary recalls; (3) age-specific general questionnaires; (4) anthropometry measurements; (5) record of calls and visits. See Supplementary Table S1 for contents of the instruments. Overall, parents or caregivers served as the main study respondents for all modules. Children aged 6–13 years were invited in varying capacities to answer questions about what they ate (Module 2) and their physical and leisure time activities (Module 3). Children aged 6–8 years assisted with the reporting, while children aged 9–13 years served as primary respondents for these sections. All modules except for the dietary recall were programmed into the Survey Solutions data collection application. The dietary recall module was collected on paper by field interviewers and then entered into the Nutrition Data System for Research (NDSR), version 2019 (University of Minnesota, Minneapolis, MN, USA) by bilingual trained nutritionists.

- Household Screener: The household screener included eligibility questions and caregiver consent for individuals eligible and willing to participate in the study. Following rostering of all members between the ages of 4 and 13 years, one child was selected randomly from each household.
- Dietary Questions and 24 h Dietary Recall. The 24 h dietary recall relied on a multiple pass approach to capture a detailed list of the foods and beverages consumed by each child. Information included the quantity consumed, preparation method (e.g., fried, boiled, added fat, sugar, salt or other ingredients); eating occasion (e.g., breakfast, lunch, dinner, snacks, beverages only); and eating location (e.g., home, someone else’s home, school, daycare, restaurant, or other). Nine hundred and eighty-three children completed a single dietary recall. Twenty-five percent of the sample from each of the three regions was randomly selected for a second 24 h dietary recall interview to estimate distributions of usual nutrient intake and to assess nutrient adequacy and excess (*n* = 250).
- Child Questionnaire. This questionnaire consisted of well-established categorical survey questions about potential influences on children’s eating patterns and food preferences to gauge the impact of nutrient intake and food choice on child health. These influences included household composition, income and economic indicator assessments, parental education level, ethnicity, race, child daycare and school participation, participation in food assistance programs, food security status, child physical and sedentary activity, sleep behaviors, screen time usage, and child health (including food allergies and food avoidance).
- Anthropometry. Measurements by trained field staff included child height, weight, and waist and hip circumferences.

### 2.3. Training

Four distinct phases of formal training were delivered. Additionally, over the course of the data collection period, supervisors provided ongoing and targeted feedback, and training to address issues discovered through routine quality assurance and quality control (QA/QC) processes and in response to concerns raised by any team member. To begin, the University of Minnesota Nutrition Coordinating Center (NCC) delivered a three-day in-person training to nutritionists from the University of São Paulo (USP) responsible for entering survey data into the NDSR and providing the first round of the QA/QC check of the data. In the second phase, over four days, study leadership and the USP nutritionists trained the field supervisors and core field interviewers assigned to collect pilot study data. In advance of the pilot data collection, study leadership and the USP nutritionists led a four-day refresher training. Field supervisors and the USP nutritionists conducted a five-day training for the full team of interviewers prior to the main data collection effort. USP nutritionists certified interview staff on dietary recall collection procedures. NCC staff certified USP nutritionists on procedures for entering the paper and pencil recalls into NDSR.

### 2.4. Pilot Study

A pilot study (*n* = 60) was conducted as a trial run of the sampling procedures, instrumentation, and associated data collection procedures, as well as the data transfer protocols among and across the full study teams. Importantly, the pilot tested data entry rules developed for the study and collected information about commonly consumed local foods to add to the NDSR food database prior to fielding the full study. Following the same sampling procedures used for the main study, pilot study interviews were collected across the three regions (20 first dietary recalls and 5 secondary dietary recalls in each).

### 2.5. Dietary Recall Collection Procedures

Following selection of households using random walk procedures, field interviewers screened households for eligibility. The interviewer then collected the 24 h dietary recall using a tailored dietary recall form and scripts aided by the Food Amount Estimation tools (Supplementary Table S2). Parents or caregivers responded on behalf of children under 6 years of age. Parents or caregivers were interviewed in the presence of children 6–8 years old, so children could provide additional information, such as foods eaten at school. Children 9–13 years old provided information about their own dietary intakes with their parent or caregiver present to provide additional details, such as preparation methods for foods prepared at home. If the child ate a meal or snack while in the care of others and the parent or caregiver did not know what the child ate, a form was used to gathering information about what their child ate during the recall day while in the care of others.

### 2.6. Dietary Recall Entry Procedures

Detailed data entry procedures were developed to ensure foods consumed in Brazil were coded using an appropriate matching food and food amount in NDSR. Food names, food specific units, and nutrient composition values for foods in NDSR are based on U.S. foods. Consequently, coding rules were determined for all foods commonly consumed by children in Brazil. In many cases a similar matching food in NDSR was found for foods as consumed in Brazil. As needed Brazilian foods (both packaged and home-prepared foods) were added to NDSR using the program’s User Recipe feature. Examples of Brazilian foods added include feijoada (black turtle beans), bolo de tapioca (creamy tapioca cake), and galinhada (a rice, chicken, and tomato dish). Food fortification differences between Brazil and the United States were considered during development of data entry procedures, and some food fortification differences (e.g., differences in grain fortification practices) were addressed via changes made to foods in the NDSR output files.

### 2.7. Questionnaire and Anthropometry Collection Procedures

Following completion of the 24 h dietary recall, interviewers administered the child questionnaire using Survey Solutions. Lastly, interviewers followed National Health and Nutrition Examination Survey procedures [15] to collect high-quality body measurements of the children using standardized examination procedures and calibrated equipment. Two interviewers worked as a team to collect and record the anthropometric data—one measured (the examiner) and the other recorded the measurements (the recorder). The examiner positioned the respondent, took all measurements, and told the recorder the measurement values to record; the recorder entered the data. All measurements were taken in duplicate.

### 2.8. Quality Assurance and Quality Control Processes

During data collection, field supervisors conducted QC checks on Survey Solutions data. USP conducted a 100% QA review on the paper recalls entered into NDSR, and NCC conducted a 10% random line-by-line QA review on recalls entered into NDSR before transferring NDSR data text files into a SAS-ready dataset for analysis.

### 2.9. Statistical Analysis

All statistical analyses, except for the descriptive statistics in Table 1 were performed on weighted data using SAS (version 9, SAS Institute Inc: Cary, NC) and SAS-callable SUDAAN^®^ (version 11, RTI International: Research Triangle Park, NC, USA) software. Data were calibrated to population totals by stratum, regional rural/urban status, and region by sex and age. We adjusted 2008 population totals in each sector by the ratio of the corresponding 2019 municipal population total and the 2008 municipal population total.

Nutrient requirements used for dietary assessment were developed and published by United States Food and Nutrition Board of the Institute of Medicine, National Academy of Sciences. All reference values and their sources are provided in Table 2; Table 3 Prevalence of inadequate nutrient intake was evaluated as the percentage of the usual intake being less than the Estimated Average Requirement (EAR). For nutrients with an Adequate Intake (AI), we calculated the percent of the population above the AI. To estimate the proportion of the population at risk of excessive intake, the outcome measure was the percentage of the population with usual intakes exceeding the Tolerable Upper Intake Level (UL). Macronutrients were compared with the Acceptable Macronutrient Distribution Range (AMDR). Before the diet could be characterized as at-risk for inadequacy or excess relative to United States Dietary Reference Intakes (DRIs) values, usual intake estimates are needed that are adjusted for random measurement error (i.e., day-to-day variation) in self-reported diets [16,17,18]. Several statistical methods are available to adjust the 24 h recall data to better estimate usual dietary intakes [19,20,21,22,23]. All of the approaches require at least two repeat measurements for a representative subsample of the population group of interest to allow computation of both variance components. For this analysis, macros developed to implement the National Cancer Institute (NCI) method [23] were used to produce the mean and standard error (SE) for a given usual intake, as well as the percentiles of intake and the probabilities of meeting the EAR, and exceeding the AI or UL, using the probability approach. The statistical models fit using this procedure incorporated the sampling weights and covariate adjustments for day of the week of the dietary recall (weekend/weekday), age group, and interview sequence (first or second dietary recall). A cutoff of an adjusted *p*-value of 0.001 was used to determine statistical significance for comparisons between regions and socioeconomic status (SES) to reduce type 1 error.

Socioeconomic status (SES) for the Brazilian population was based on methodology developed by the Brazilian Economic Classification Criteria [24]. Questionnaire responses were used to determine point values for each respondent based on 12 domestic comfort items in the household, educational level of the head of the household, and the household’s access to public utility services. Based on the total sum of the points for each item, each respondent was linked to one of the six SES groups. For this analysis, groups were further combined into the following: A, B1, and B2 were combined to denote a ‘high’ SES. C1 and C2 were combined to denote “moderate” SES, while respondents in the D and E categories were combined for “low” SES.

## 3. Results

A total of 983 children participated in the study, 516 younger children (4–8 years old) and 467 older children (9–13 years old) (Table 1). The sample was distributed across the geographical regions of the Northeast, Southeast, and South. Approximately half of the sample was from moderate SES, with 30% from low SES, and 20% from high SES. Three-quarters of parents/caregivers were married or from households with partners, and 83% were the biological mothers. The proportion by race was not different between age groups (data not shown).

### 3.1. Nutrient Intakes by Age

*Younger children (4–8 years).* Mean energy intakes among younger children were 1522 kcal/day (SE = 14) (Table 2). Approximately 36.5% of younger children exceeded 10% of their daily energy from saturated fat. Only 22.3% met the WHO recommendation for <10% of daily energy from added sugars, but nearly all were below the 25% cut-off used by the US. No young children met the EAR for vitamin D (100%) and a majority did not meet the EARs for vitamins E (78.1%) and calcium (80.5%). Nearly one-in-five fell short of achieving the EAR for vitamin A. Similarly, their intakes of fiber and potassium were low when compared with the recommendations (AI). Two-thirds (67.1%) consumed sodium at levels above the UL.

*Older children (9–13 years).* Mean energy intakes among older children were 1719 kcal/day (SE = 17) (Table 3). Approximately one-third of older children exceeded 10% of their daily energy from saturated fat. Like the younger children, only 22.5% were below the WHO recommendations of 10% of daily energy from added sugars, but few exceeded the guidelines of 25% from the US (1.2%). As with the younger children, a large proportion of older children had inadequate nutrient intakes for vitamin D (100%), vitamin E (96.5%), and calcium (97.7%). Additionally, a majority of older children consumed inadequate amounts of phosphorus (75.0%) and vitamin A (71.6%). Furthermore, nearly half consumed too little magnesium (45.9%) and more than a fifth had too little vitamin C (21.5%). Similarly, older children’s intakes of fiber, potassium, and choline were low compared with recommendations, while consumption of sodium (76.3%) exceeded the UL.

### 3.2. Nutrient Intakes by Region (4–13 Years)

Mean energy intake was 1595 kcal/day (SE = 16) in the Northeast, 1601 kcal/day (SE = 20) in the Southeast, and 1720 kcal/day (SE = 25) in the South (Table 4). The Northeast had the highest percentage of children under the AMDR for fat (59.3% vs. 10.7% in the Southeast and 6.2% in the South). Saturated fat intakes were highest in the South (20 g/day ± 0.4), followed by the Southeast (17 g/day ± 0.2), and were lowest in the Northeast (15 g/day ± 0.2). The Northeast had the lowest percentage of children exceeding AI for vitamin K (6.4%), followed by the South (47.6%), and then the Southeast (65.1%). Although potassium percentage exceeding the AI was low in general, the South had more children above the AI than both the Northeast and the Southeast (16.7% vs. 2.9% and 5.8%). The same pattern was found for calcium with inadequacy in the South 80.4%, compared to 90.2% in the Northeast, and 93.1% in the Southeast. The South had the highest inadequacy of Vitamin C (38.1%) compared to both Northeast (10.4%) and the Southeast (13.6%). Pairwise *p*-values for the comparisons by region can be found in Table 5.

### 3.3. Nutrient Intake by SES (4–13 Years)

Low SES households had a higher percentage of children with low energy intakes from fat (% below the AMDR) (50.9% vs. 26.0% Moderate and 15.0% High SES) (Table 6). Saturated fat intakes were highest among the high SES group (19.0 g/day ± 0.2) compared to the moderate and low SES groups (17.1 g/day ± 0.2 and 15.4 g/day ± 0.2, respectively), resulting in nearly half of the children in the high SES groups exceeding 10% of their daily energy from saturated fat. For added sugars, 31.6% of children in low SES households were below the WHO recommendations for added sugars, versus 14.9% in moderate and 17.1% in high SES households. The low SES households had the lowest percentage of children with inadequate intake of magnesium (15.1%) compared to moderate (27.1%) and high (28.5%) SES households. The high SES group had the lowest percentage of intakes above the AI for potassium (1.1%) compared to 8.4% and 7.8% in the low and moderate SES groups. High-SES households had the highest percentage exceeding AI for choline (50.0%) when compared to the low and moderate-SES (18.1% and 17.1%, respectively). Overall, other nutrient intakes between the SES groups were similar. Pairwise *p*-values for nutrient intake distributions by SES can be found in Table 7.

## 4. Discussion

Childhood diets shape lifelong food preferences and health outcomes [21]. Suboptimal diets are a major contributor to chronic diseases, including diabetes, heart disease, stroke, cancer, and obesity over a lifetime, contributing to significant morbidity and premature mortality [22]. In childhood, inadequate micronutrient intake is particularly concerning because children have higher nutrient requirements by body weight due to their developmental needs and their still immature immune systems and physiologic behaviors [26,27]. The objectives of this sizable national study were to learn about the nutrient intakes and gaps of children in the three most populated regions of Brazil during a time when dietary habits are established and to examine how intakes are affected by SES and regional factors. Studies that evaluate the nutrient consumption of Brazilian children are essential to guide public policies to mitigate inadequacies.

In terms of macronutrients, few children consumed inadequate amounts of carbohydrates and protein. In our study, more than 30% of children in both of the age ranges reported fat consumption below the AMDR and saturated fat above the UL. Our results align with Bueno et al. [6] who reported macronutrient intakes within the acceptable range except for total fat in children aged 4 to 6 years of age, where 23% of children of this age reported total fat consumption below the AMDR, and 30% reported saturated fat levels greater than the WHO recommendations.

Although the intakes of B-vitamins generally met recommendations, we identified inadequate intakes of vitamin D, vitamin E, calcium, and dietary fiber among school-aged children in Brazil. In addition, we observed more inadequacies in the diets of older children (9–13 years) compared to younger children (4–8 years), notably for vitamin C, phosphorus, and magnesium. Bueno et al. (2013) evaluated the nutrient consumption of children aged 2–6 years in nine Brazilian cities. Similar to our findings, among children aged 4–6 years, few were inadequate in B-vitamins, while consumption of fiber, calcium, and vitamins D and E was lower than recommended, and sodium exceeded recommendations. Like our study, the Brazilian 2017–2018 Households Expenditure Survey (in Portuguese, *Pesquisa de Orçamentos Familiares* or POF) study reported a high prevalence of inadequacy for vitamin D, vitamin E, and calcium among 10–18-year-olds [28]. The Brazilian Study of Cardiovascular Risks in Adolescents (ERICA 2013–2014) [29] evaluated adolescents aged 12–17 years and also reported a high inadequacy for vitamin E and calcium, and excessive levels of sodium, showing persistent issues with intakes of these nutrients in children and adolescents in Brazil. When evaluating the vitamin D findings, it is important to consider that Brazil is primarily a tropical country and vitamin D can be synthesized by the sun when exposed to UV- β radiation. However, irregular and inadequate sun exposure impedes cutaneous vitamin D synthesis. Self-reported food intake does not capture sun-induced vitamin D synthesis [30].

Studies have reported that there is an adaptation of our palates to salty and sweet tastes during childhood that may be transferred into adulthood [31,32]. We found that fewer than a quarter of Brazilian children met the WHO recommendations for less than 10% of daily energy from added sugars [33]. In older children and adolescents, the POF reported sugar intakes averaging 59 g/day for males and 52 g/day for females [28], which is considerably higher than the amounts observed in the present study. Similarly, ERICA showed added sugars exceeding the maximum recommended limit. This is not unexpected, because it is well-documented that intakes of added sugars increase with age [34,35]. Excessive consumption of added sugars have been associated with overweight in children and the early development of chronic noncommunicable diseases such as diabetes and hypertension.

Similarly, excess sodium intake is a problem in Brazil [36]. While iodine deficiency decreased after salt fortification, higher than recommended sodium intake is now reported because of the inclusion of large amounts of sodium in food products. Our findings are similar to those from the ERICA study, where excessive sodium was reported for 84–91% of children 12–13 years of age [4], as well as reports of excess sodium consumption shown in studies of younger children [6,30]. Excessive consumption of sodium by children impacts blood pressure and can increase the risk of cardiovascular diseases [37], besides being associated with foods associated with weight gain and childhood obesity [38].

There are few large-scale Brazilian nutrient intake studies of children spanning the ages of 4–13 years across different geographical regions. A review of Brazilian dietary intake studies among young children (6 months to 6 years) from 2005 to 2015 identified 31 studies, of which only three concurrently assessed children from different geographical regions and 23 included mainly quantitative data [30]. Unlike our findings, among the younger children studied in this review, calcium requirements were generally achieved or even exceeded, while like our results, sodium intake was usually higher. Because of the lack of recent data, it will be important in the future to examine the dietary patterns leading to differences in nutrient intakes by region.

Low socioeconomic status (SES) has been associated with inadequate nutrient intakes and poor adherence to dietary guidance [39,40]. Our study showed that almost all children across SES categories had energy intakes within the AMDRs for carbohydrates and protein. However, across all SES categories, close to one-third had energy intakes outside of the AMDR for fat, with half of children from low-SES households below the AMDR, putting them at risk for inadequate essential fatty acid intakes. In contrast, the percentage of energy from saturated fat exceeding the AMDR increased with SES, with nearly half of children from high-SES households exceeding the recommendations. Added sugars intake was lowest among children from the lowest SES group. Monteiro et al. [41] recently reported on the quality of the Brazilian population’s diet and observed that adolescents, females, and individuals with high incomes were more likely to have lower quality diets.

Our study has many strengths. The sampling plan ensured broad representation of the population in three geographically diverse regions of the country. This study is representative of 83% of the population in the country. Detailed dietary intake information and second day recalls for a sub-set of participants allowed for the estimation of usual nutrient intakes. The study is not without limitations, however. Because food-based dietary guidelines aim to encourage adequate nutrient intake from the diet, our estimates do not include supplements. While we did collect the names of all supplements consumed during the same 24 h period as the dietary recall, only 7.4% of our sample reported consuming dietary supplements, so the impact of these missing sources of nutrients is likely to be small. Another limitation is that there are no nutrient recommendations for children in Brazil, so we estimated adequacy based on the standards set for the United States. Other researchers in Brazil have used a similar approach when evaluating diet adequacy, including the Brazilian study of food consumption—POF [6,28,42,43,44,45,46,47,48]. Furthermore, while most of our results aligned with those presented in comparison studies, the divergence may be attributable to dietary intake methodology, or the age groups assessed. Limited large studies of dietary intake in children with geographical diversity exist in Brazil.

## 5. Conclusions

The contrast observed between the diets of young Brazilians and dietary recommendations underscores the need for individual and environmental interventions to facilitate healthier dietary intake patterns. Healthy eating in childhood and adolescence is important for proper growth and development and to prevent various health conditions, including high blood pressure, heart disease, type 2 diabetes, cancer, osteoporosis, iron deficiency, and dental caries [49]. Our data suggests that among all children, it is difficult to obtain recommended intakes of vitamin D, vitamin E, and calcium from the current selection of food and beverages. Concern for the balance of types of fats consumed by all children, but particularly those in the lowest and highest SES groups, is warranted, with concomitant low overall energy intakes from total fat and excessive percentages of energy intakes from saturated fat.

## Figures and Tables

**Table 1:** Characteristics of Brazilian children 4–13 years of age and their household demographics ^a^.

Characteristic	All Ages		4–8 Years		9–13 Years	
	Sample Size	Percentage (SE)	Sample Size	Percentage (SE)	Sample Size	Percentage (SE)
**Child’s Sex**						
Male	512	52 ± 1.6	270	52 ± 2.2	242	52 ± 2.3
Female	471	48 ± 1.6	246	48 ± 2.2	225	48 ± 2.3
**Region**						
Northeast	334	34 ± 1.5	169	33 ± 2.1	165	35 ± 2.2
Southeast	331	34 ± 1.5	185	36 ± 2.1	146	31 ± 2.2
Southern	318	32 ± 1.5	162	31 ± 2.0	156	33 ± 2.2
**Household Socioeconomic Status**						
Low	291	30 ± 1.5	157	30 ± 2.0	134	29 ± 2.1
Moderate	500	51 ± 1.6	267	52 ± 2.2	233	50 ± 2.3
High	192	20 ± 1.3	92	18 ± 1.7	100	21 ± 1.9
**Caregiver’s Sex**						
Male	127	13 ± 1.1	50	10 ± 1.3	77	16 ± 1.7
Female	856	87 ± 1.1	466	90 ± 1.3	390	84 ± 1.7
**Caregiver’s Marital Status**						
Married	396	42 ± 1.6	209	42 ± 2.2	187	42 ± 2.3
Separated or divorced	78	8 ± 0.9	39	8 ± 1.2	39	9 ± 1.3
Widowed	35	4 ± 0.6	14	3 ± 0.7	21	5 ± 1.0
Never married	122	13 ± 1.1	62	12 ± 1.5	60	13 ± 1.6
Living with partner	317	33 ± 1.5	175	35 ± 2.1	142	32 ± 2.2
**Caregiver’s Highest Level of Education**						
No/Incomplete School	97	10 ± 1.0	39	8 ± 1.2	58	13 ± 1.6
Elementary	212	22 ± 1.4	98	20 ± 1.8	114	25 ± 2.1
Jr. High School	199	21 ± 1.3	120	24 ± 1.9	79	18 ± 1.8
High School	370	39 ± 1.6	201	40 ± 2.2	169	38 ± 2.3
Higher Education	70	7 ± 0.9	41	8 ± 1.2	29	6 ± 1.2
**Other**						
Government Benefits Participation	370	38 ± 1.6	198	38 ± 2.1	172	37 ± 2.2
Own Home	684	70 ± 1.5	348	67 ± 2.1	336	72 ± 2.1
Biological Mother	710	83 ± 1.3	387	83 ± 1.7	323	83 ± 1.9

^a^ Sample data in this table is unweighted.

**Table 2:** Usual energy and nutrient intake distributions in foods and beverages for children aged 4–8 years (*n* = 516).

	DRI Value ^a,b^			Intake		DRI Compliance (%) ^a,b^		
Nutrient	EAR/AMDR	AI	UI	Mean	SE	<EAR/AMDR	>AI	>UL/AMDR
**Macronutrients**								
Energy (kcal/day)	-	-	-	1522	14	-	-	-
Fat (g/day)	-	-	-	46.6	0.5	-	-	-
Saturated Fat (g/day)	-	-	-	16.0	0.2	-	-	-
Added Sugars (g/day)	-	-	-	39.7	0.9	-	-	-
Carbohydrate (g/day)	130	-	-	217.8	2.5	4.0%	-	-
Protein (g/day)	19	-	-	61.4	0.6	0.0%	-	-
Dietary Fiber (g/day)	-	25	-	14.2	0.2	-	2.3%	-
Fat (% kcal)	25–35	-	-	27.3	0.2	33.2%	-	6.0%
Saturated Fat (% kcal) ^c^	-	-	10	9.4	0.1	-	-	36.5%
Added Sugars (% kcal) ^d^	-	-	10 or 25	13.6	0.2	22.3%	-	1.2%
Total Sugar (% kcal)	-	-	-	22.1	0.2	-	-	-
Carbohydrate (% kcal)	45–65	-	-	57.3	0.3	2.2%	-	9.1%
Protein (% kcal)	10–30	-	-	16.3	0.1	0.2%	-	0.0%
**Micronutrients**								
Vitamin A (μg RAE/day) ^e^	275	-	900	441.2	8.7	19.8%	-	0.0%
Thiamin (mg/day)	0.5	-	-	1.1	0.01	0.1%	-	-
Riboflavin (mg/day)	0.5	-	-	123.4	3.18	0.0%	-	-
Niacin (mg/day) ^f^	6	-	15.0	22.8	0.6	1.5%	-	-
Vitamin B-6 (mg/day)	0.5	-	40	1.3	0.01	0.0%	-	0.0%
Folate (μg DFE/day) ^f^	160	-	400	393.0	4.9	0.2%	-	0.0%
Vitamin B-12 (μg/day)	1.0	-	-	3.4	0.05	0.0%	-	-
Choline (mg/day)	-	250	1000	234.0	1.9	-	32.6%	0.0%
Vitamin C (mg/day)	22	-	650	152.7	8.6	8.0%	-	2.6%
Vitamin D (μg/day)	10	-	75	2.3	0.1	100.0%	-	0.0%
Vitamin E (mg/day) ^f^	6	-	300	4.9	0.07	78.1%	-	0.0%
Vitamin K (μg/day)	-	55	-	47.4	0.8	-	28.2%	-
Calcium (mg/day)	800	-	2500	610.5	10.7	80.5%	-	0.0%
Iron (mg/day)	4	-	40	11.3	0.2	0.1%	-	0.0%
Magnesium (mg/day)	110	-	110	194.9	2.1	1.5%	-	-
Phosphorus (mg/day)	405	-	3000	877.3	8.9	0.1%	-	0.0%
Potassium (mg/day)	-	2300	-	1687	18.9	-	8.7%	-
Sodium (mg/day)	-	1000	1900	2204	25.5	-	99.8%	67.1%
Zinc (mg/day)	4.0	-	12	8.8	0.07	0.0%	-	4.3%

AI, Adequate Intake; DFE, Dietary Folate Equivalent; DRI, Dietary Reference Intake; EAR, Estimated Average Requirement; RAE, retinol activity equivalent; SE, Standard Error; UL, Tolerable Upper Intake Level. ^a^ All DRIs are from *Dietary reference intakes summary tables* [25], ^b^ Unless otherwise indicated, means + SEs, or percentages of DRI compliance based on usual intakes derived from the National Cancer Institute method. Micronutrient intakes do not include dietary supplements. When the DRI is a range, the DRI compliance value in the < column is percent below the lower end of the range, and the value in the > column is percent above the higher end of the range. ^c^ Saturated fat intake should be as low as possible while consuming a nutritionally adequate diet. For the purposes of this evaluation, we used 10% of daily energy as the maximum. ^d^ The WHO recommends limiting added sugars to 10% of daily energy, while the US NASEM recommends no more than 25% of daily energy. The value in the <EAR is associated with the % below the WHO recommendation; whereas the amount shown in >UL is % above the US NASEM recommendation, ^e^ Vitamin A is reported as RAE, and only the UL is based on retinol (preformed vitamin A), ^f^ Percentage above the UL is based on the consumption of the synthetic form of the vitamin only.

**Table 3:** Usual energy and nutrient intake distributions in foods and beverages for children aged 9–13 years (*n* = 467).

	DRI Value ^a,b^			Intake		DRI Compliance (%) ^a,b^		
Nutrient	EAR/AMDR	AI	UI	Mean	SE	<EAR/AMDR	>AI	> UL/AMDR
**Macronutrients**								
Energy (kcal/day)	-	-	-	1719	17	-	-	-
Fat (g/day)	-	-	-	52.9	0.6	-	-	-
Saturated Fat (g/day)	-	-	-	17.6	0.2	-	-	-
Added Sugars (g/day)	-	-	-	43.9	1.0	-	-	-
Carbohydrate (g/day)	130	-	-	243.9	2.9	1.3%	-	-
Protein (g/day)	34	-	-	68.7	0.7	0.2%	-	-
Dietary Fiber (g/day)	-	F: 26 M: 31	-	16.0	0.2	-	0.8%	-
Fat (% kcal)	25–35	-	-	27.4	0.2	32.2%	-	6.2%
Saturated Fat (% kcal) ^c^	-	-	10	9.2	0.1	-	-	32.6%
Added Sugars (% kcal) ^d^	-	-	10 or 25	13.5	0.2	22.5%	-	1.3%
Total Sugar (% kcal)	-	-	-	19.9	0.2	-	-	-
Carbohydrate (% kcal)	45–65	-	-	57.0	0.3	2.5%	-	8.4%
Protein (% kcal)	10–30	-	-	16.2	0.1	0.2%	-	0.0%
**Micronutrients**								
Vitamin A (μg RAE/day) ^e^	F: 420 M: 445	-	1700	378.0	7.9	71.6%	-	0.0%
Thiamin (mg/day)	0.7	-	-	1.1	0.01	2.2%	-	-
Riboflavin (mg/day)	0.8	-	-	144.5	3.66	0.0%	-	-
Niacin (mg/day) ^f^	9	-	20.0	25.4	0.7	4.2%	-	-
Vitamin B-6 (mg/day)	0.8	-	60	1.5	0.02	0.7%	-	0.0%
Folate (μg DFE/day) ^f^	250	-	600	445.4	5.7	2.9%	-	0.0%
Vitamin B-12 (μg/day)	1.5	-	-	3.3	0.05	1.6%	-	-
Choline (mg/day)	-	375	2000	257.0	2.2	-	1.4%	0.0%
Vitamin C (mg/day)	39	-	1200	145.3	8.8	21.5%	-	0.4%
Vitamin D (μg/day)	10	-	100	2.1	0.1	100.0%	-	0.0%
Vitamin E (mg/day) ^f^	9	-	600	5.3	0.08	96.5%	-	0.0%
Vitamin K (μg/day)	-	60	-	56.2	0.9	--	36.1%	-
Calcium (mg/day)	1100	-	3000	550.7	10.3	97.7%	-	0.0%
Iron (mg/day)	F: 5.7 M: 5.9	-	40	12.7	0.2	1.3%	-	0.0%
Magnesium (mg/day)	200	-	350	210.8	2.4	45.9%	-	1.2%
Phosphorus (mg/day)	1055	-	4000	925.3	9.9	75.0%	-	0.0%
Potassium (mg/day)	-	F: 2300 M: 2500	-	1763	20.7	-	6.3%	-
Sodium (mg/day)	-	1200	2200	2699	31.0	-	99.9%	76.3%
Zinc (mg/day)	7.0	-	23	9.8	0.09	5.2%	-	0.0%

AI, Adequate Intake; DFE, Dietary Folate Equivalent; DRI, Dietary Reference Intake; EAR, Estimated Average Requirement; F, females; M, males; RAE, retinol activity equivalent; SE, Standard Error; UL, Tolerable Upper Intake Level. ^a^ All DRIs are from *Dietary reference intakes summary tables* [25], ^b^ Unless otherwise indicated, means + SEs, or percentages of DRI compliance based on usual intakes derived from the National Cancer Institute method. Micronutrient intakes do not include dietary supplements. When the DRI is a range, the DRI compliance value in the < column is percent below the lower end of the range, and the value in the > column is percent above the higher end of the range, ^c^ Saturated fat intake should be as low as possible while consuming a nutritionally adequate diet. For the purposes of this evaluation, we used 10% of daily energy as the maximum, *^d^* The WHO recommends limiting added sugars to 10% of daily energy, while the US NASEM recommends no more than 25% of daily energy. The value in the <EAR is associated with the % below the WHO recommendation; whereas the amount shown in >UL is % above the US NASEM recommendation, ^e^ Vitamin A is reported as RAE, and only the UL is based on retinol (preformed vitamin A), ^f^ Percentage above the UL is based on the consumption of the synthetic form of the vitamin only.

**Table 4:** Usual energy and nutrient intake distributions in foods and beverages for children aged 4–13 years (*n* = 983) by region.

	Northeast (*n* = 334)				Southeast (*n* = 331)				South (*n* = 318)			
	Intake	DRI Compliance (%) ^a,b^			Intake	DRI Compliance (%) ^a,b^			Intake	DRI Compliance (%) ^a,b^		
Nutrient	Mean ± SE	<EAR/AMDR	>AI	>UL/AMDR	Mean ± SE	<EAR/AMDR	>AI	>UL/AMDR	Mean ± SE	<EAR/AMDR	>AI	>UL/AMDR
**Macronutrients**												
Energy (kcal/day)	1595 ± 16	-	-	-	1601 ± 20	-	-	-	1720 ± 25	-	-	-
Fat (g/day)	43.3 ± 0.6	-	-	-	52.3 ± 0.3	-	-	-	59.0 ± 1.0	-	-	-
Saturated Fat (g/day)	15.1 ± 0.2	-	-	-	17.1 ± 0.2	-	-	-	20.1 ± 0.4	-	-	-
Added Sugars (g/day)	40.0 ± 1.0	-	-	-	42.0 ± 1.2	-	-	-	45.0 ± 1.4	-	-	-
Carbohydrate (g/day)	237.4 ± 3.0	0.7%	-	-	224.7 ± 3.5	4.7%	-	-	229.6 ± 3.8	3.9%	-	-
Protein (g/day)	66.3 ± 0.6	0.0%	-	-	61.4 ± 0.8	0.4%	-	-	70.3 ± 1.1	0.2%	-	-
Dietary Fiber (g/day)	15.4 ± 0.3	-	1.2%	-	14.9 ± 0.3	-	1.4%	-	15.2 ± 0.3	-	3.1%.	-
Fat (% kcal)	24.2 ± 0.3	59.3%	-	1.4%	29.2 ± 0.2	10.7%	-	5.0%	30.1 ± 0.2	6.2%	-	8.9%
Saturated Fat (% kcal) ^c^	8.5 ± 0.1	-	-	23.8%	9.6 ± 0.1	-	-	39.0%	10.3 ± 0.1	-	-	51.7
Added Sugars (% kcal)^d^	13.3 ± 0.2	24.5%	-	1.0%	13.6 ± 0.2	22.4%	-	1.5%	14.0 ± 0.2	16.1%	-	1.0%
Total Sugar (% kcal)	21.0 ± 0.3	-	-	-	20.8 ± 0.3	-	-	-	21.5 ± 0.2	-	-	-
Carbohydrate (% kcal)	59.5 ± 0.3	1.1%	-	17.6%	56.1 ± 0.3	1.3%	-	3.8%	54.0 ± 0.3	6.9%	-	2.4%
Protein (% kcal)	16.9 ± 0.1	0.0%	-	0.0%	15.5 ± 0.1	0.2%	-	0.0%	16.6 ± 0.1	0.0%	-	0.0%
**Micronutrients**												
Vitamin A (μg RAE/day)^e^	441.8 ± 15.5	46.9%	-	0.0%	406.9 ± 4.9	40.5%	-	0.0%	342.2 ± 9.0	58.0%	-	0.0%
Thiamin (mg/day)	1.1 ± 0.01	0.0%	-	-	1.0 ± 0.01	4.5%	-	-	1.1 ± 0.02	2.2%	-	-
Riboflavin (mg/day)	110.3 ± 4.02	0.0%	-	-	149.6 ± 4.24	0.0%	-	-	151.6 ± 3.98	0.0%	-	-
Niacin (mg/day) ^f^	41.6 ± 1.3	0.1%	-	92.1%	13.5 ± 0.2	3.4%	-	16.1%	17.5 ± 0.2	0.0%	-	47.2%
Vitamin B-6 (mg/day)	1.4 ± 0.01	0.0%	-	0.0%	1.4 ± 0.02	0.2%	-	0.0%	1.5 ± 0.03	2.1%	-	0.0%
Folate (μg DFE/day) ^f^	446.8 ± 5.5	0.0%	-	0.0%	395.8 ± 6.3	3.8%	-	0.0%	407.5 ± 6.7	3.3%	-	0.0%
Vitamin B-12 (μg/day)	3.2 ± 0.07	2.0%	-	-	3.3 ± 0.05	0.3%	-	-	3.6 ± 0.06	0.2%	-	-
Choline (mg/day)	247.3 ± 1.3	-	0.0%	0.0%	237.8 ± 2.0	-	16.3%	0.0%	257.4 ± 4.5	-	26.7%	0.0%
Vitamin C (mg/day)	323.7 ± 31.6	10.4%	-	8.5%	82.2 ± 3.2	13.6%	-	0.0%	45.8 ± 1.8	38.1%	-	0.0%
Vitamin D (μg/day)	2.4 ± 0.1	99.8%	-	0.0%	2.0 ± 0.1	100.0%	-	0.0%	2.2 ± 0.1	100.0%	-	0.0%
Vitamin E (mg/day) ^f^	4.7 ± 0.09	90.7%	-	0.0%	5.3 ± 0.08	87.3%	-	0.0%	5.6 ± 0.11	80.1%	-	0.0%
Vitamin K (μg/day)	36.8 ± 0.7	-	6.4%	-	63.4 ± 0.6	-	65.1%	-	59.2 ± 1.1	-	47.6%	-
Calcium (mg/day)	556.6 ± 13.2	90.2%	-	0.0%	567.8 ± 9.8	93.1%	-	0.0%	662.4 ± 18.0	80.4%	-	0.0%
Iron (mg/day)	13.5 ± 0.2	0.0%	-	0.0%	10.8 ± 0.2	2.4%	-	0.0%	11.1 ± 0.2	0.7%	-	0.0%
Magnesium (mg/day)	204.8 ± 2.1	14.5%	-	0.2%	196.0 ± 2.8	29.1%	-	1.0%	212.9 ± 3.3	23.3%	-	2.3%
Phosphorus (mg/day)	898.0 ± 8.8	37.9%	-	0.0%	867.9 ± 10.6	41.8%	-	0.0%	982.8 ± 15.6	30.3%	-	0.0%
Potassium (mg/day)	1683 ± 19.2	-	2.9%	-	1701 ± 21.9	-	5.8%	-	1866 ± 31.2	-	16.7%	-
Sodium (mg/day)	2337 ± 34.1	-	99.9%	66.6%	2482 ± 35.8	-	99.9%	73.3%	2647 ± 41.1	-	99.9%	80.3%
Zinc (mg/day)	9.0 ± 0.04	0.0%	-	0.0%	8.8 ± 0.12	9.1%	-	3.2%	11.0 ± 0.17	1.5%	-	12.2%

AI, Adequate Intake; DFE, Dietary Folate Equivalent; DRI, Dietary Reference Intake; EAR, Estimated Average Requirement; F, females; M, males; RAE, retinol activity equivalent; SE, Standard Error; UL, Tolerable Upper Intake Level, ^a^ All DRIs are from *Dietary reference intakes summary tables* [25], ^b^ Unless otherwise indicated, means + SEs, or percentages of DRI compliance based on usual intakes derived from the National Cancer Institute method. Micronutrient intakes do not include dietary supplements. When the DRI is a range, the DRI compliance value in the < column is percent below the lower end of the range, and the value in the > column is percent above the higher end of the range, ^c^ Saturated fat intake should be as low as possible while consuming a nutritionally adequate diet. For the purposes of this evaluation, we used 10% of daily energy as the maximum, ^d^ The WHO recommends limiting added sugars to 10% of daily energy, while the US NASEM recommends no more than 25% of daily energy. The value in the <EAR is associated with the % below the WHO recommendation; whereas the amount shown in >UL is % above the US NASEM recommendation, ^e^ Vitamin A is reported as RAE, and only the UL is based on retinol (preformed vitamin A), ^f^ Percentage above the UL is based on the consumption of the synthetic form of the vitamin only.

**Table 5:** Pairwise *p*-values for usual energy and nutrient intake distributions in foods and beverages for children aged 4–13 years (*n* = 983) by region.

	Northeast vs. Southeast			Northeast vs. South			Southeast vs. South		
	DRI Compliance (%) ^a,b^			DRI Compliance (%) ^a,b^			DRI Compliance (%) ^a,b^		
Nutrient	<EAR/AMDR	>AI	>UL/AMDR	<EAR/AMDR	>AI	>UL/AMDR	<EAR/AMDR	>AI	>UL/AMDR
**Macronutrients**									
Energy (kcal/day)	-	-	-	-	-	-	-	-	-
Fat (g/ day)	-	-	-	-	-	-	-	-	-
Saturated Fat (g/ day)	-	-	-	-	-	-	-	-	-
Added Sugars (g/ day)	-	-	-	-	-	-	-	-	-
Carbohydrate (g/ day)	0.001	-	-	0.005	-	-	0.601	-	-
Protein (g/ day)	0.226	-	-	0.441	-	-	0.551	-	-
Dietary Fiber (g/ day)	-	0.794	-	-	0.086	-	-	0.142	-
Fat (% kcal)	0.000	-	0.268	0.000	-	0.015	0.039	-	0.073
Saturated Fat (% kcal)	-	-	0.000	-	-	0.000	-	-	0.001
Added Sugars (% kcal)	0.520	-	0.886	0.007	-	0.995	0.041	-	0.884
Total Sugar (% kcal)	-	-	-	-	-	-	-	-	-
Carbohydrate (% kcal)	0.832	-	0.000	0.000	-	0.000	0.000	-	0.345
Protein (% kcal)	0.637	-	1.000	0.835	-	1.000	0.536	-	1.000
**Micronutrients**									
Vitamin A	0.095	-	1.000	0.004	-	1.000	0.000	-	1.000
Thiamin	0.000	-	-	0.007	-	-	0.106	-	-
Riboflavin	-	-	-	-	-	-	-	-	-
Niacin	0.001	-	0.000	0.750	-	0.000	0.001	-	0.000
Vitamin B-6	0.398	-	1.000	0.007	-	1.000	0.022	-	1.000
Folate	0.000	-	1.000	0.001	-	0.864	0.738	-	1.000
Vitamin B-12	0.048	-	-	0.032	-	-	0.773	-	-
Choline	-	0.000	1.000	-	0.000	1.000	-	0.001	1.000
Vitamin C	0.203	-	0.001	0.000	-	0.007	0.000	-	1.000
Vitamin D	0.368	-	1.000	0.377	-	1.000	-	-	-
Vitamin E	0.164	-	1.000	0.000	-	1.000	0.012	-	1.000
Vitamin K	-	0.000	-	-	0.000	-	-	0.000	-
Calcium	0.168	-	1.000	0.000	-	0.994	0.000	-	0.994
Iron	0.004	-	1.000	0.128	-	1.000	0.077	-	1.000
Magnesium	0.000	-	0.809	0.004	-	0.496	0.092	-	0.704
Phosphorus	0.299	-	1.000	0.043	-	1.000	0.002	-	1.000
Potassium	-	0.067	-	-	0.000	-	-	0.000	-
Sodium	-	0.936	0.000	-	0.931	0.000	-	0.868	0.000
Zinc	0.000	-	0.042	0.022	-	0.000	0.000	-	0.000

AI, Adequate Intake; DRI, Dietary Reference Intake; EAR, Estimated Average Requirement; F, females; M, males; UL, Tolerable Upper Intake Level, ^a^ All DRIs are from Dietary reference intakes summary tables [25], ^b^ DRI compliance based on usual intakes derived from the National Cancer Institute method.

**Table 6:** Usual energy and nutrient intake distributions in foods and beverages for children aged 4–13 years (*n* = 983) by socioeconomic status.

	DE (Low) (*n* = 291)				C (Moderate) (*n* = 500)				A-B (High) (*n* = 192)			
	Intake	DRI Compliance (%) ^a,b^			Intake	DRI Compliance (%) ^a,b^			Intake	DRI Compliance (%) ^a,b^		
Nutrient	Mean ± SE	<EAR/AMDR	>AI	>UL/AMDR	Mean ± SE	<EAR/AMDR	>AI	>UL/AMDR	Mean ± SE	<EAR/AMDR	>AI	>UL/AMDR
**Macronutrients**												
Energy (kcal/day)	1636 ± 20	-	-	-	1601 ± 20	-	-	-	1633 ± 17	-	-	-
Fat (g/day)	46.5 ± 0.7	-	-	-	52.3 ± 0.3	-	-	-	54.9 ± 0.6	-	-	-
Saturated Fat (g/day)	15.4 ± 0.2	-	-	-	17.1 ± 0.2	-	-	-	19.0 ± 0.2	-	-	-
Added Sugars (g/day)	39.7 ± 1.2	-	-	-	42.0 ± 1.2	-	-	-	42.5 ± 1.6	-	-	-
Carbohydrate (g/day)	240.9 ± 3.4	0.7%	-	-	224.7 ± 3.5	5.0%	-	-	220.5 ± 3.5	1.5%	-	-
Protein (g/day)	65.9 ± 1.0	0.1%	-	-	61.4 ± 0.8	0.0%	-	-	66.5 ± 0.8	0.0%	-	-
Dietary Fiber (g/day)	16.5 ± 0.3	-	2.5%	-	14.9 ± 0.3	-	1.3%.	-	14.0 ± 0.3	-	0.4%	-
Fat (% kcal)	25.2 ± 0.3	50.9%	-	3.1%	29.2 ± 0.2	26.0%	-	5.0%	29.7 ± 0.3	15.0%	-	13.0%
Saturated Fat (% kcal) ^c^	8.4 ± 0.1	-	-	18.1%	9.6 ± 0.1	-	-	38.9%	10.2 ± 0.2	-	-	48.8%
Added Sugars (% kcal) ^d^	13.0 ± 0.3	31.6%	-	2.3%	13.6 ± 0.2	14.9%	-	0.3%	14.4 ± 0.3	17.1%	-	1.9%
Total Sugar (% kcal)	19.8 ± 0.4	-	-	-	20.8 ± 0.3	-	-	-	22.6 ± 0.3	-	-	-
Carbohydrate (% kcal)	58.9 ± 0.4	1.6%	-	15.6%	56.1 ± 0.3	1.1%	-	5.9%	54.8 ± 0.4	5.7%	-	2.8%
Protein (% kcal)	16.4 ± 0.2	0.4%	-	0.0%	15.5 ± 0.1	0.0%	-	0.0%	16.3 ± 0.2	0.4%	-	0.0%
**Micronutrients**												
Vitamin A (μgRAE/day) ^e^	432.9 ± 15.4	46.4%	-	0.0%	406.9 ± 4.9	53.4%	-	0.0%	489.1 ± 4.9	34.0%	-	0.0%
Thiamin (mg/day)	1.1 ± 0.02	1.3%	-	-	1.0 ± 0.01	1.9%	-	-	1.1 ± 0.01	0.0%	-	-
Riboflavin (mg/day)	134.5 ± 5.43	0.0%	-	-	149.6 ± 4.24	0.0%	-	-	119.5 ± 4.37	0.0%	-	-
Niacin (mg/day) ^f^	31.5 ± 1.4	2.9%	-	71.7%	13.5 ± 0.2	2.8%	-	57.4%	18.8 ± 0.4	0.2%	-	54.6%
Vitamin B-6 (mg/day)	1.4 ± 0.02	0.1%	-	0.0%	1.4 ± 0.02	0.8%	-	0.0%	1.5 ± 0.02	0.0%	-	0.0%
Folate (μg DFE/day) ^f^	456.2 ± 8.3	0.6%	-	0.0%	395.8 ± 6.3	3.9%	-	0.0%	399.8 ± 1.1	0.0%	-	0.0%
Vitamin B-12 (μg/day)	3.3 ± 0.10	6.2%	-	-	3.3 ± 0.05	0.1%	-	-	4.3 ± 0.05	0.0%	-	-
Choline (mg/day)	256.7 ± 3.2	-	18.1%	0.0%	237.8 ± 2.0	-	17.1%	0.0%	256.0 ± 1.3	-	50.0%	0.0%
Vitamin C (mg/day)	232.9 ± 8.9	21.5%	-	4.7%	82.2 ± 3.2	8.7%	-	0.6%	75.2 ± 3.8	16.8%	-	0.0%
Vitamin D (μg/day)	2.1 ± 0.1	99.9%	-	0.0%	2.0 ± 0.1	100.0%	-	0.0%	2.5 ± 0.1	99.8%	-	0.0%
Vitamin E (mg/day) ^f^	5.0 ± 0.09	91.2%	-	0.0%	5.3 ± 0.08	84.7%	-	0.0%	5.3 ± 0.07	89.2%	-	0.0%
Vitamin K (μg/day)	46.6 ± 1.3	-	24.4%	-	63.4 ± 0.6	65.0%	35.0%	-	56.5 ± 1.1	-	42.9%	-
Calcium (mg/day)	524.5 ± 14.1	91.8%	-	0.0%	567.8 ± 9.8	89.2%	-	0.0%	661.9 ± 17.0	84.8%	-	0.0%
Iron (mg/day)	12.7 ± 0.2	0.0%	-	0.0%	10.8 ± 0.2	1.9%	-	0.0%	11.2 ± 0.2	0.1%	-	0.0%
Magnesium (mg/day)	212.6 ± 2.9	15.1%	-	2.0%	196.0 ± 2.8	27.1%	-	1.2%	195.4 ± 2.2	28.5%	-	0.0%
Phosphorus (mg/day)	899.0 ± 13.2	35.6%	-	0.0%	867.9 ± 10.6	39.8%	-	0.0%	949.2 ± 4.0	37.5%	-	0.0%
Potassium (mg/day)	1735± 27.2	-	8.4%	-	1701 ± 21.9	-	7.8%	-	1763 ± 18.2	-	1.1%	-
Sodium (mg/day)	2498 ± 47.0	-	99.6%	69.9%	2482± 35.8	-	99.7%	67.5%	2444 ± 28.9	-	100.0%	100.0%
Zinc (mg/day)	8.9 ± 0.11	4.7%	-	1.6%	8.8 ± 0.12	1.0%	-	1.4%	9.9 ± 0.10	0.0%	-	0.6%

AI, Adequate Intake; DFE, Dietary Folate Equivalent; DRI, Dietary Reference Intake; EAR, Estimated Average Requirement; F, females; M, males; RAE, retinol activity equivalent; SE, Standard Error; UL, Tolerable Upper Intake Level, ^a^ All DRIs are from *Dietary reference intakes summary tables* [25], ^b^ Unless otherwise indicated, means + SEs, or percentages of DRI compliance based on usual intakes derived from the National Cancer Institute method. Micronutrient intakes do not include dietary supplements. When the DRI is a range, the DRI compliance value in the < column is percent below the lower end of the range, and the value in the > column is percent above the higher end of the range, ^c^ Saturated fat intake should be as low as possible while consuming a nutritionally adequate diet. For the purposes of this evaluation, we used 10% of daily energy as the maximum, ^d^ The WHO recommends limiting added sugars to 10% of daily energy, while the US NASEM recommends no more than 25% of daily energy. The value in the <EAR is associated with the % below the WHO recommendation; whereas the amount shown in >UL is % above the US NASEM recommendation, ^e^ Vitamin A is reported as RAE, and only the UL is based on retinol (preformed vitamin A), ^f^ Percentage above the UL is based on the consumption of the synthetic form of the vitamin only.

**Table 7:** Pairwise *p*-values for usual energy and nutrient intake distributions in foods and beverages for children aged 4–13 years (*n* = 983) by SES.

	Low vs. Moderate			Low vs. High			Moderate vs. High		
	DRI Compliance (%) ^a,b^			DRI Compliance (%) ^a,b^			DRI Compliance (%) ^a,b^		
Nutrient	<EAR/AMDR	>AI	>UL/AMDR	<EAR/AMDR	>AI	>UL/AMDR	<EAR/AMDR	>AI	>UL/AMDR
**Macronutrients**									
Energy (kcal/ day)	-	-	-	-	-	-	-	-	-
Fat(g/ day)	-	-	-	-	-	-	-	-	-
Saturated fat(g/ day)	-	-	-	-	-	-	-	-	-
Added Sugars (g/ day)	-	-	-	-	-	-	-	-	-
Carbohydrate (g/ day)	0.001	-	-	0.362	-	-	0.038	-	-
Protein (g/ day)	0.763	-	-	0.645	-	-	0.756	-	-
Dietary Fiber (g/ day)	-	0.209	-	-	0.081	-	-	0.317	-
Fat (% kcal)	0.000	-	0.569	0.000	-	0.018	0.002	-	0.026
Saturated Fat (% kcal)	-	-	0.000	-	-	0.000	-	-	0.009
Added Sugars (% kcal)	0.000	-	0.497	0.000	-	0.908	0.479	-	0.617
Total Sugar (% kacal)	-	-	-	-	-	-	-	-	-
Carbohydrate (% kcal)	0.613	-	0.000	0.012	-	0.000	0.000	-	0.014
Protein (% kcal)	0.232	-	1.000	0.914	-	1.000	0.197	-	1.000
**Micronutrients**									
Vitamin A	0.059	-	1.000	0.006	-	1.000	0.000	-	1.000
Thiamin	0.513	-	-	0.118	-	-	0.056	-	-
Riboflavin	0.851	-	-	0.908	-	-	-	-	-
Niacin	0.906	-	0.000	0.031	-	0.000	0.034	-	0.021
Vitamin B-6	0.191	-	1.000	0.649	-	1.000	0.206	-	1.000
Folate	0.005	-	1.000	0.304	-	1.000	0.006	-	1.000
Vitamin B-12	0.000	-	-	0.000	-	-	0.688	-	-
Choline	-	0.720	1.000	-	0.000	1.000	-	0.000	1.000
Vitamin C	-	-	0.093	0.207	-	0.205	0.002	-	0.840
Vitamin D	0.545	-	1.000	0.774	-	1.000	0.364	-	1.000
Vitamin E	-	-	1.000	0.485	-	1.000	0.124	-	1.000
Vitamin K	-	0.002	-	-	0.000	-	-	0.055	-
Calcium	0.236	-	1.000	0.016	-	1.000	0.113	-	1.000
Iron	0.020	-	0.972	0.934	-	1.000	0.061	-	0.977
Magnesium	0.000	-	0.782	0.000	-	0.583	0.700	-	0.755
Phosphorus	0.238	-	1.000	0.668	-	1.000	0.577	-	1.000
Potassium	-	0.771	-	-	0.001	-	-	0.001	-
Sodium	-	0.914	0.000	-	0.393	0.000	-	0.425	0.000
Zinc	0.001	-	0.892	0.003	-	0.536	0.202	-	0.262

AI, Adequate Intake; DRI, Dietary Reference Intake; EAR, Estimated Average Requirement; F, females; M, males; UL, Tolerable Upper Intake Level, ^a^ All DRIs are from Dietary reference intakes summary tables [25], ^b^ DRI compliance based on usual intakes derived from the National Cancer Institute method.

## Supplementary Materials

The following are available online at https://www.mdpi.com/article/10.3390/nu14030485/s1, Table S1: Instrument Modules used in the Brazil Kids Nutrition and Health Study, Table S2: Food amount estimation kit inventory and guide for use in the Brazil Kids Nutrition and Health Study.
