# Supplementary material for: Nutrient Intakes among Brazilian Children Need Improvement and Show Differences by Region and Socioeconomic Level
Source: Nutrients. 2022 Jan 22;14(3):485. doi: 10.3390/nu14030485 (PMC8837958; doi:10.3390/nu14030485)
Supplement: Supplementary file 1 [file nutrients-14-00485-s001.zip › nutrients-1525353-supplementary.pdf]

Supplementary Table S1. Instrument Modules used in the Brazil Kids Nutrition and Health Study.

Module	Name	Data Elements	
1	Household Screener	Eligibility criteria and child roster<br>Caregiver Consent	Location information about the household
2	Dietary Questions and 24-hr Dietary Recall (separate modules for 2 age groups and 1st and 2nd 24-hr recalls)	Child assent<br>Foods, beverages and dietary supplements consumed<br>Quantities of foods, beverages and dietary supplements<br>Location of foods consumed	Eating occasions<br>Foods fed by other adults (if applicable)<br>Normalcy of diet prior day
3	Child Questionnaire	Socio-demographics of child and household<br>Feeding practices<br>Physical activity and free time activity levels<br>Sleep patterns<br>Screen time<br>Water type	Food allergies<br>Foods avoided<br>Childcare/school<br>Food security<br>Organic food choices<br>Special diets
4	Anthropometry	Weight, height, arm, waist and hip circumference	
5	Record of Calls, Appointment Scheduling	Status of each contact attempt at household	Appointment reminders to return to the household

Supplementary Table S2. Food amount estimation kit inventory and guide for use in the Brazil Kids Nutrition and Health Study.

Tool	Guide for Use
**Food Amount Estimation Booklet**[1]	
Grid for Squares and Rectangles	Non-loaf bread, cake
Circles	Biscuits, salty pancakes, meatballs
Wedges	Pies, cake, pizza
Height	Cheese, non-loaf bread, cake
Butter on knives	Butter, jam
Mounds	Rice, rice containing dishes, beans, bean containing dishes, mashed cassava, sweet potatoes, ice cream, pudding, pasta/noodles, and similar shaped foods
Fish	Fish fillets
Chicken	Type of chicken piece and fraction consumed
**Tableware**[1]	
Copo de requeijao: cream cheese cup	Cold beverages (e.g., water, milk, juice)
Copo americano: American cup (small, large)	Cold beverages (e.g., water, milk, juice)
Xicara de cha: cup for tea	Hot beverages (e.g., tea, cocoa)
Xicara de cafe: cup for coffee	Hot beverages (e.g., tea, cocoa)
Colher de sopa: tablespoon	Condiments, sauces, sugar
Colher de sobremesa: dessert spoon	Condiments, sauces, sugar
Colher de cha: teaspoon	Condiments, sauces, sugar
Colher de cafe: coffee spoon	Condiments, sauces, sugar
Bowls (2 sizes)	Soups, cereals
**Plastic food replicas**	
Roast beef	Portion
Hamburger patties (2 sizes)	Small and large patty

[1] Food portions could be estimated using the visual tools in the Food Estimation booklet (e.g., grids, circles, wedges, etc.) which corresponded to amounts in the Nutrition Data System for Research. Similarly, the model tableware (e.g., cups, spoons and bowls) were of known volume and could be used to estimate portions consumed. Colored lines indicated different volumes if the full amount was not consumed.
